# Low-cost Imaging of Fluorescent DNA in Agarose Gel Electrophoresis using Raspberry Pi cameras

**DOI:** 10.1007/s10895-021-02884-0

**Published:** 2022-01-22

**Authors:** Hassan Ali Abid, Jian Wern Ong, Eric Shen Lin, Zhixiong Song, Oi Wah Liew, Tuck Wah Ng

**Affiliations:** 1 Laboratory for Optics, Department of Mechanical & Aerospace Engineering, Monash University, & Mechanics, AcousticsClayton, VIC 3800 Australia; 2 Centre for Translational Medicine, Cardiovascular Research Institute, Yong Loo Lin School of Medicine, National University of Singapore, National University Health System, 14 Medical Drive, 117599 Singapore

**Keywords:** Gel electrophoresis, Agarose, Deoxyribonucleic acid, Polymerase Chain Reaction, Imaging

## Abstract

Low-cost analytical solutions built around microcomputers like the Raspberry Pi help to facilitate laboratory investigations in resource limited venues. Here, three camera modules (V1.3 with and without filter, as well as NoIR) that work with this microcomputer were assessed for their suitability in imaging fluorescent DNA following agarose gel electrophoresis. Evaluation of their utility was based on signal-to-noise (SNR) and noise variance metrics that were developed. Experiments conducted with samples were subjected to Polymerase Chain Reaction (PCR), and the amplified products were separated using gel electrophoresis and stained with Midori green. Image analysis revealed the NoIR camera performed the best with SNR and noise variance values of 21.7 and 0.222 respectively. In experiments conducted using UV LED lighting to simulate ethidium bromide (EtBr) excitation, the NoIR and V1.3 with filter removed cameras showed comparable SNR values.

## Introduction

Gel electrophoresis has been an integral part of molecular biology labs for decades and continues to be vital tool in the analytical laboratory [1–3]. Two of most common matrices used in gel electrophoresis for nucleic acid separation are agarose and polyacrylamide. Although agarose gels have a lower resolving power compared with polyacrylamide gels, they are able to resolve DNA fragments over a wider size range. Agarose of the appropriate pore size can be used to separate fragments ranging between 50 to 20,000 base pairs (bp) [4, 5]. The DNA fragments loaded on agarose gels have traditionally been stained using ethidium bromide (EtBr) and detected via ultraviolet (UV) transilluminator systems [6]. Free and DNA-bound EtBr absorbs strongly at ultraviolet wavelengths of 286 and 270 nm with highest emission at 605 nm [7]. DNA binding induces a small blue-shift of the excitation maxima and 40-fold increase in fluorescence intensity with respect to free EtBr. EtBr is however a highly mutagenic agent [8, 9] and even shown to alter the agarose electrophoretic mobility of some DNA structures [10]. This has motivated the development of alternative non-mutagenic fluorescent stains such as SYBR-Green, SYBR-Gold, and Midori-Green [11–13]. Midori-Green has excitation peaks in the ultraviolet (270 and 290 nm) and blue-green (490 nm) wavelength region with an emission peak centered at 530 nm.

The imaging aspect of electrophoresis gels has not received much developmental attention although this has a large influence on fluorescent measurement sensitivity [14]. There is now strong interest to develop low-cost analytical solutions in the laboratory due to the increasing availability of affordable electronic modules [14, 15]. The Raspberry Pi microcomputer has been widely adopted as soon as it was launched in 2012 due to its low cost while still possessing sufficiently high levels of processing capabilities [16]. This microcomputer has an onboard connector which, through a detachable ribbon cable is able to receive data communication from a low-cost camera module. One version of the camera, commonly referred to as the V1.3, has a sensor (Omnivision OV5647) that captures 10-bit raw image data at a resolution of 2592 × 1944 pixels and has been used in scientific imaging applications [17, 18]. As this sensor has a native sensitivity that extends beyond the visible range, removing the attached Bayer filter (resulting in the attenuation of wavelengths outside of the visible range) has been shown to permit imaging in the UV spectrum [18]. Another version of the camera, known as the NoIR, records 10-bit raw image data at a resolution of 3280 × 2464 pixels. There is no filter attached to this camera’s sensor (Sony IMX219) and it has been used in scientific imaging applications [19, 20].

**Fig. 1:**
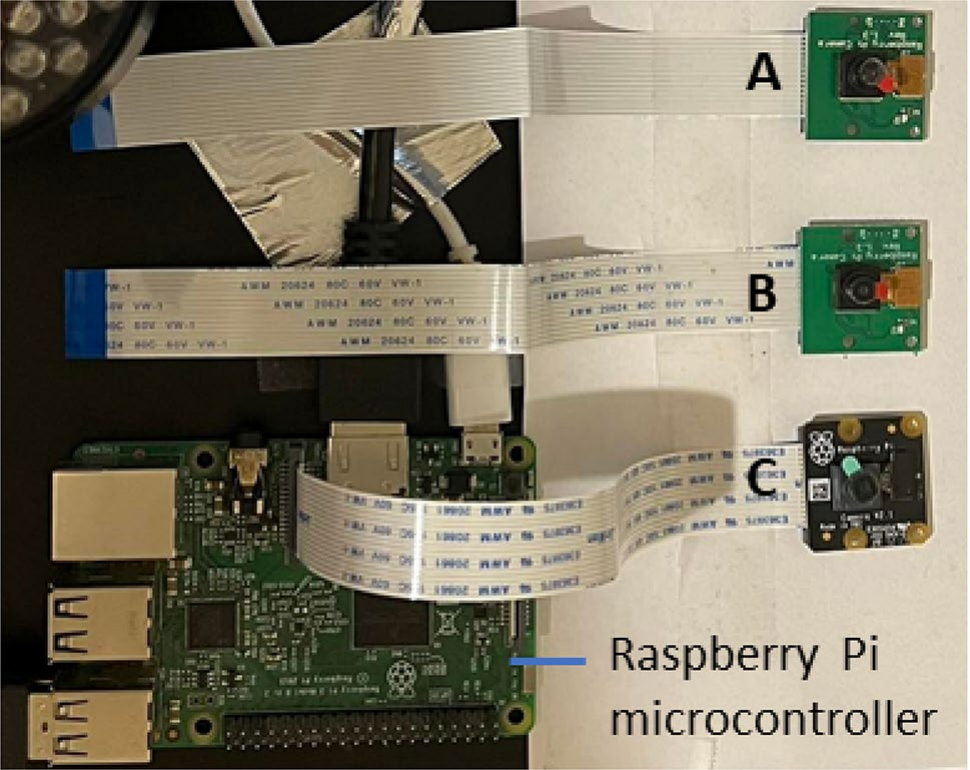
The three Raspberry Pi cameras, the (**A**) V1.3, (**B**) V1.3 with filter removed, and (**C**) NoIR, used to interface with the Raspberry Pi microcontroller to conduct the investigation

In this work, the performances of three Raspberry Pi cameras, the V1.3 with filter, the V1.3 without filter, and the NoIR interfaced with a Raspberry Pi microcontroller (see Fig. 1) to image fluorescent DNA fragments separated by gel electrophoresis were investigated. The images recorded were analyzed using algorithms that provided quantitative measures of the signal-to-noise ratios (SNR) and the noise variances.

## Materials and Methods

Modification of the V1.3 camera was done by first removing the lens from the board. Following this, the sensor was peeled from the board and the filter separated from the sensor, both with the help of a scalpel blade. The filter-free sensor was then attached back onto the board and the lens affixed after that. The other V1.3 and NoIR cameras were used as is without modification for the experiments.

The DNA samples used were obtained from a kit (miniPCR, KT-1010–01). The reaction setup was prepared in 4 different vials; N – a negative control, P – a positive control, and two environmental DNA samples A and B. Each contained 10 µL of the template DNA combined with 10 µL of the PARE primer mix, 5 µL of the 5X EZ PCR master mix, and 2 µL of the Midori Green stain. The reaction samples from each vial was transferred to 75 □μl capillary glass tubes (Hirschmann) that were sealed at both ends using putty. The tubes were then loaded in a tilting platform thermal cycler system for PCR thermal cycling amplification [21]. The PCR protocol parameters applied for thermal cycling are given in Table 1. Upon completion of thermal cycling, the samples were extracted from the capillary tubes using a bulb dispenser (Drummond) to undergo gel electrophoresis.

**Table 1:** The protocol used to perform PCR on the DNA samples

	Initial Denaturation	Denaturation	Annealing	Extension	Final Extension
Temperature	94 °C	94 °C	55 °C	72 °C	72 °C
Time	30 s	10 s	10 s	10 s	30 s
Number of Cycles = 28					

To resolve the amplified DNA fragments, 1% agarose gel was prepared by weighing 0.2 g of agarose powder (Scientifix, 901E) using an electronic balance and added to a glass flask containing 20 ml of 1X Tris/Borate/EDTA (TBE) buffer with pH 8.3. The mixture was microwave heated for 25 s and swirled to facilitate mixing. The solution was left to cool for 2 min at room temperature. The flask was swirled again to ensure good mixing of the Midori Green stain with the agarose solution. The solution was then poured into the blueGel™ (miniPCR) gel casting tray and comb was inserted for wells creation. The solution was left for 20 min to allow for cooling and solidification of the gel. Following this, the comb was removed and the gel transferred to the blueGel™ (miniPCR) electrophoresis chamber which was filled with 30 ml of 1X TBE buffer to ensure the gel was completely submerged. Each well was filled with 10 □μl of the 100 bp DNA ladder and amplified PCR products/samples were loaded into the wells of gel. Following this, the gel was run for 40 min and imaged using the Raspberry Pi cameras.

The recorded intensity *f* at each picture element (pixel) location (denoted by *i* and *j*) of the stained gel segments in each image can be taken to be the sum of the signal *s* and noise *n* or

1 $$f(i,j)=s(i,j)+n(i,j)$$

The variances in the signal and noise can be defined respectively as

2 $${\sigma }_{s}^{2}=\langle {\left|s\left(i,j\right)-\langle s\left(i,j\right)\rangle \right|}^{2}\rangle$$

3 $${\sigma }_{n}^{2}=\langle {\left|n\left(i,j\right)-\langle n\left(i,j\right)\rangle \right|}^{2}\rangle$$

Since the signal to noise ratio (SNR) is defined in terms of the standard deviation of signal and noise or

4 $$SNR=\frac{{\sigma }_{s}}{{\sigma }_{n}}$$

the SNR can be practically determined from the recorded intensity *f* and the noise *n* from the background using

5 $$SNR=\sqrt{\frac{{\sigma }_{f}^{2}}{{\sigma }_{n}^{2}}-1}$$

In order to determine the SNR metric in Eq. (4) software routines were written using the C +  + programming language in conjunction with the Cool Image (CImg) template image processing toolkit. It should be noted that it was necessary to do this by first splitting the image into its red, green and blue color components. The SNR of each of them was computed separately, with the final value of the SNR being the average of the three components.

The sensitivity of the cameras towards UV light was tested by placing them 5 cm away from a UV LED light source built with 56 LEDs, each with 350 mcd luminous intensity and 30° beam angle. Images of the light source were recorded as the voltage supplied to light source was progressively increased from 0 to 16 V. From these images, the SNR was determined as in the previous case.

## Results and Discussion

**Fig. 2:**
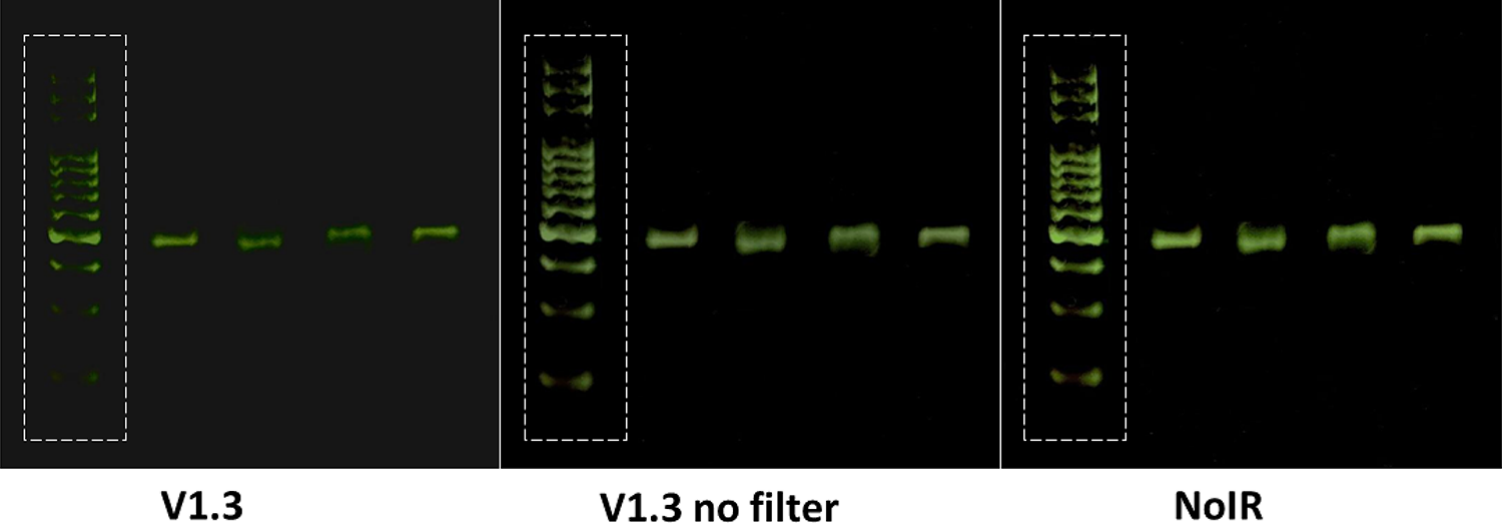
The DNA samples imaged using the V1.3, V1.3 without filter and NoIR Raspberry Pi cameras following electrophoresis via the blueGel™ chamber. The signal-to-noise ratio was calculated from the area covered by the white dashed lines

The images of the fluorescent DNA samples separated using electrophoresis recorded using the 3 cameras are shown in Fig. 2. From visual examination, there were visible intensity differences of the stained bands against the background in each of the images.

The outcomes of the quantitative analysis conducted are summarized in Table 2. It can be seen that removing the filter from the V1.3 camera improved the average SNR by 1.89 times. However, the improvement using the NoIR camera was higher at 2.29 times. It is noteworthy that this improvement was mainly attained through the red (2.43 times) and green (2.81 times) channels. Yet, the noise variance in the red, green, and blue channels are roughly invariant for this camera. In comparing the V1.3 cameras, there was reduced noise variance in all channels when the filter was removed. As in the NoIR camera, the SNR improvement was attained primarily through the red (2.04 times) and green (2.41 times) channels. These results demonstrate how differently the signals from each of the camera sensors are processed into the red, green and blue color components.

**Table 2:** Summary of signal to noise ratios (SNR) and noise variance values calculated from the stained gels separated by electrophoresis using the three Raspberry Pi cameras

	**V1.3**	**V1.3 (with filter removed)**	**NoIR**
*SNR*			
*Red channel*	9.34379	19.0196	22.7153
*Green channel*	10.1762	24.5448	28.5398
*Blue channel*	8.92380	10.1443	13.9505
*Average*	9.48126	17.9029	21.7352
*Noise variance*			
*Red channel*	2.77993	0.461046	0.224453
*Green channel*	2.78468	0.489464	0.235608
*Blue channel*	2.76844	0.413587	0.206740
*Average*	2.777683	0.454699	0.222267

**Fig. 3:**
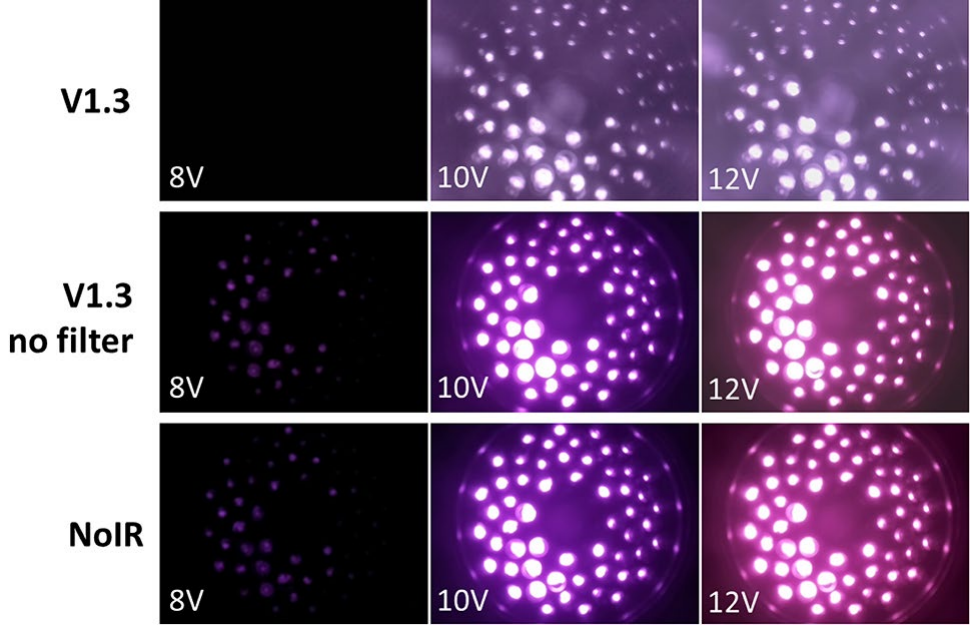
Images of the UV LED array light source supplied at 8 V, 10 V and 12 V and recorded using V1.3, V1.3 without filter and NoIR Raspberry Pi cameras. The signal-to-noise ratio was calculated from the entire image

**Fig. 4:**
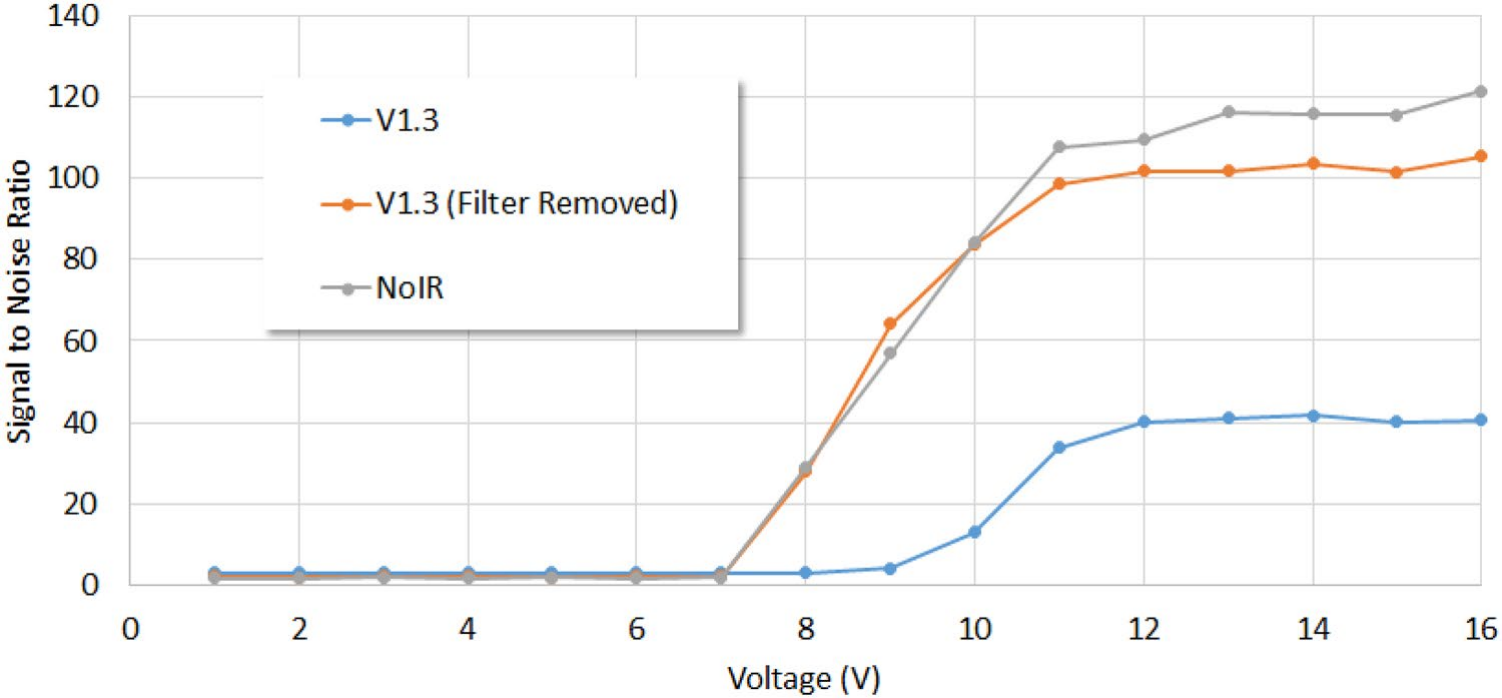
Plots of the signal-to-noise ratios found using the V1.3, V1.3 without filter and NoIR Raspberry Pi cameras against the voltages applied to drive the UV LED light source

The Midori green stain used here had a primary excitation peak at 490 nm as well as two secondary peaks at 270 nm and 290 nm. Hence, this stain is compatible with both UV and blue LED transilluminators for excitation. On the other hand, DNA-bound EtBr is optimally excited at 250 nm [7] and this will require the use of light sources operating solely in the UV region. The UV LED light source experiment was conducted to simulate the performance of EtBr gel staining of DNA electrophoresis gels. From the images recorded using the 3 Raspberry Pi cameras Fig. 3, it can be seen that the NoIR and V1.3 without filter cameras had markedly higher brightness than the V1.3 camera. The results from computing the SNR are presented in Fig. 4. It is evident that all the cameras only began detecting output from the light source when the driving voltage exceeded 7 V. In addition, the output from the light source reached its saturation maximum when the driving voltage was above 12 V. It is also clear that SNR for the NoIR and the V1.3 without filter cameras were roughly the same until the driving voltage reached 10 V, wherein the former had marginally higher values when the driving voltage exceeded that. These results indicated that the NoIR and V1.3 without filter cameras can be expected to have similar performances when used for DNA gel electrophoresis imaging with EtBr staining. It should be noted that, apart from the toxicity with this stain, there are also phototoxic (actinic) effects posed to users when UV transilluminators are used for prolonged viewing [22].

**Fig. 5:**
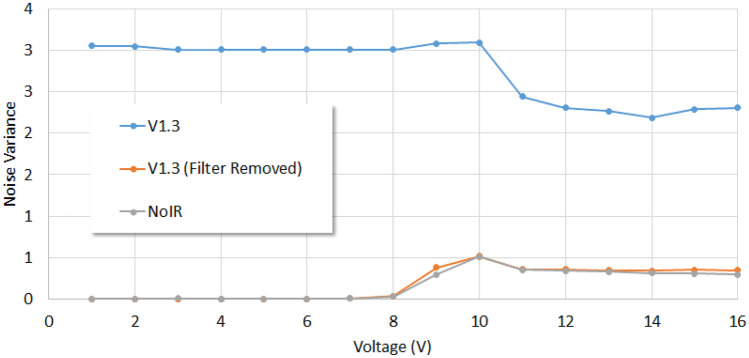
Plots of the noise variances determined using the V1.3, V1.3 without filter and NoIR Raspberry Pi cameras against the voltages applied to drive the UV LED light source

When the cameras were analyzed for noise variances (see Fig. 5), the NoIR and V1.3 without filter cameras demonstrated almost verbatim results. That the values were close to zero below 7 V driving voltage (when no output was emitted by the light source) is a typical characteristic found in most cameras. Interestingly, the noise variance of the V1.3 camera was non-zero when no output was emitted from the light source. This is attributed to the Bayer filter (which appears as a mosaic of color filters) that is placed over the sensor of this camera. The graphics processing unit (GPU) in the Raspberry Pi microcomputer implements a demosaicing algorithm on the signal from each picture element (pixel) in order to yield a color image. These algorithms are, however, not immune to sensor noise [23] which is shown here to be exacerbated when low light levels are encountered. Raspberry Pi cameras with Bayer filters should hence be carefully assessed when used for the scientific imaging of low light level events.

It is apt to note that the application of Raspberry Pi microcomputers with cameras offers the advantage of dedicated operation notwithstanding the increasing adoption of smartphones for scientific analysis [24, 25]. Coupled with the ease of interfacing a multitude of other devices (e.g. actuators, sensors, etc.) to the microcomputer, this approach offers a better capacity to build up laboratory automation solutions.

## Conclusions

It has been found that Raspberry Pi cameras, despite their relatively low costs, are able to provide measurements of fluorescent DNA samples following electrophoresis. Their direct connection to the Raspberry Pi offers the ability to develop turnkey instrumentation that is suited for use in low resource venues. The NoIR model provided the highest SNR among the cameras, and together with the obviation of need for any careful filter removal (that might damage the sensor), makes it most feasible for use in this application. It is envisaged that with the proper adaptation, these Raspberry Pi cameras can be incorporated into the instrumentation that conducts thermal cycling of DNA samples. This will then yield the information that relates amplification reaction rates and times to the relative and absolute amounts of DNA present in PCR at any time.

## Data Availability

All data generated or analyzed during this study are included in this published article.

## Code Availability

Code used in this work is written in C +  + . It is available by writing to the corresponding author.
